# Cross-Reactivity and Anti-viral Function of Dengue Capsid and NS3-Specific Memory T Cells Toward Zika Virus

**DOI:** 10.3389/fimmu.2018.02225

**Published:** 2018-10-01

**Authors:** Mei Qiu Lim, Emmanuelle A. P. Kumaran, Hwee Cheng Tan, David C. Lye, Yee Sin Leo, Eng Eong Ooi, Paul A. MacAry, Antonio Bertoletti, Laura Rivino

**Affiliations:** ^1^Programme in Emerging Infectious Diseases, Duke-NUS Medical School, Singapore, Singapore; ^2^Immunology Programme, Department of Microbiology and Immunology, Life Science Institute, National University of Singapore, Singapore, Singapore; ^3^Communicable Disease Centre, Institute of Infectious Disease and Epidemiology, Tan Tock Seng Hospital, Singapore, Singapore; ^4^Lee Kong Chian School of Medicine, Nanyang Technological University, Singapore, Singapore; ^5^Yong Loo Lin School of Medicine, National University of Singapore, Singapore, Singapore; ^6^National Centre for Infectious diseases, NCID, Singapore, Singapore; ^7^Singapore Immunology Network, Singapore Agency for Science, Technology and Research (A^*^STAR), Singapore, Singapore

**Keywords:** dengue virus (DENV), Zika virus (ZIKV), T cells, cross-reactive immune response, heterologous immunity, anti-viral response

## Abstract

Zika virus (ZIKV), a flavivirus with homology to dengue virus (DENV), is spreading to areas of DENV hyper-endemicity. Heterologous T cell immunity, whereby virus-specific memory T cells are activated by variant peptides derived from a different virus, can lead to enhanced viral clearance or diminished protective immunity and altered immunopathology. In mice, CD8+ T cells specific for DENV provide *in vivo* protective efficacy against subsequent ZIKV infection. In humans, contrasting studies report complete absence or varying degrees of DENV/ZIKV T cell cross-reactivity. Moreover, the impact of cross-reactive T cell recognition on the anti-viral capacity of T cells remains unclear. Here, we show that DENV-specific memory T cells display robust cross-reactive recognition of ZIKV NS3 *ex vivo* and after *in vitro* expansion in respectively *n* = 7/10 and *n* = 9/9 dengue-immune individuals tested. In contrast, cross-reactivity toward ZIKV capsid is low or absent. Cross-reactive recognition of DENV or ZIKV NS3 peptides elicits similar production of the anti-viral effector mediators IFN-γ, TNF-α, and CD107a. We identify 9 DENV/ZIKV cross-reactive epitopes, 7 of which are CD4+ and 2 are CD8+ T cell epitopes. We also show that cross-reactive CD4+ and CD8+ T cells targeting novel NS3 epitopes display anti-viral effector potential toward ZIKV-infected cells, with CD8+ T cells mediating direct lyses of these cells. Our results demonstrate that DENV NS3-specific memory T cells display anti-viral effector capacity toward ZIKV, suggesting a potential beneficial effect in humans of pre-existing T cell immunity to DENV upon ZIKV infection.

DENV is transmitted through the bite of an infected *Aedes* mosquito and infects an estimated 390 million people each year (1). ZIKV, a flavivirus with homology to DENV, shares the *Aedes* mosquito vector and co-exists in the same geographical areas as DENV (2). Infection with DENV or ZIKV may cause asymptomatic infection or a diverse spectrum of clinical manifestations. DENV infection may lead to uncomplicated dengue fever (DF) or the life-threatening dengue hemorrhagic fever (DHF), or dengue shock syndromes (DSS), which are characterized by increased capillary permeability and ipovolemic shock (1). ZIKV infection causes clinical symptoms that resemble but are generally milder to those caused by DENV. However, in recent outbreaks ZIKV infection has been associated with severe neurological complications such as Guillain–Barré syndrome (GBS) in adults (3) and congenital birth defects including microcephaly in fetuses born to ZIKV-infected mothers (4–7). Similar neurological complications have been reported to occur in infant non-human primates infected with ZIKV after birth (8). These findings indicate that ZIKV is a major emerging public health concern. The co-circulation of DENV and ZIKV and the recent availability of a vaccine against DENV (9) raise the need to understand the impact of pre-existing immunity to DENV on the immune-recognition of ZIKV. Recent studies have shown that human DENV E protein-reactive antibodies cross-react with ZIKV but are poorly neutralizing and instead potently enhance ZIKV infection *in vitro*, through a mechanism termed antibody dependent enhancement (10, 11). One of these studies addresses whether cross-reactive T cells could play a similar detrimental role upon ZIKV infection, however no evidence of cross-reactive CD4+ T cell recognition between DENV and ZIKV envelope and NS1 proteins is found (10). This data is in contrast with previous findings that report cross-reactive CD4+ T cell recognition between distinct flaviviruses (12–14). A more recent study shows cross-reactive recognition between DENV and ZIKV NS3 helicase, but not NS3 protease, based on a IFN-γ ELISPOT (15). Moreover, the impact of cross-reactive recognition on T cell functionality toward ZIKV is not defined. Another recent study reports the presence of DENV/ZIKV cross-reactive CD4+ and CD8+ T cells in *n* = 4/5 and *n* = 2/6 dengue seropositive individuals, respectively, and in individuals who had received a live attenuated dengue vaccine (16). Importantly, this work shows that prior immunity to dengue leads to a more vigorous T cell response to ZIKV infection with T cells expressing higher levels of the activation/exhaustion marker PD-1 and granzyme B.

Studies in mice associate cross-reactive T cell recognition with enhanced T cell immunity and improved viral clearance and/or immunopathology (17). According to the “original antigenic sin” hypothesis pre-existing memory T cells can exert a detrimental role upon either secondary infection of a closely related virus (e.g., with a different DENV serotype) (18) or upon infection of an unrelated virus that shares T cell epitopes with the primary-infecting virus (19). Immune-pathology may occur because of the suboptimal activation of cross-reactive T cells which results in a cytokine profile that is skewed toward production of pro-inflammatory cytokines, such as TNF-α over that of anti-viral mediators (20–24), or suboptimal cytotoxicity but high cytokine producing capacity (25). However, a number of recent studies support a protective rather than a detrimental effect of cross-reactive T cells during secondary dengue infection (26–29) or during ZIKV infection of dengue-immune animals (30–32). Depletion of DENV-ZIKV cross-reactive CD8+ T cells leads to increased viral burden in mice. These studies in immunocompromised mouse models strongly support the view of a protective role of cross-reactive T cells, however they may not fully recapitulate what occurs in immune competent humans. Therefore, studies in humans are needed.

In the current study we investigate the extent and functional impact in dengue-immune subjects of T cell cross-reactivity toward ZIKV capsid and NS3. We show that recognition of ZIKV NS3 is robust while that of ZIKV capsid is low or absent. Cross-reactive recognition of ZIKV peptides elicits similar cytokine profiles (IFN-γ, TNF-α, and CD107a) as those observed in the presence of DENV peptides, demonstrating lack of skewing of the T cell response toward heterologous peptides. In peptide-specific T cell lines we show that cross-reactive CD4+ and CD8+ T cells display anti-viral effector potential toward ZIKV-infected cells and that CD8+ T cells can efficiently lyse ZIKV-infected cells. These data support findings from the mouse studies and point toward a potential beneficial role of dengue-specific cross-reactive T cells during ZIKV infection.

## Results

### DENV-immune individuals display *ex vivo* T cell cross-reactivity toward ZIKV peptides

**Figure 1:**
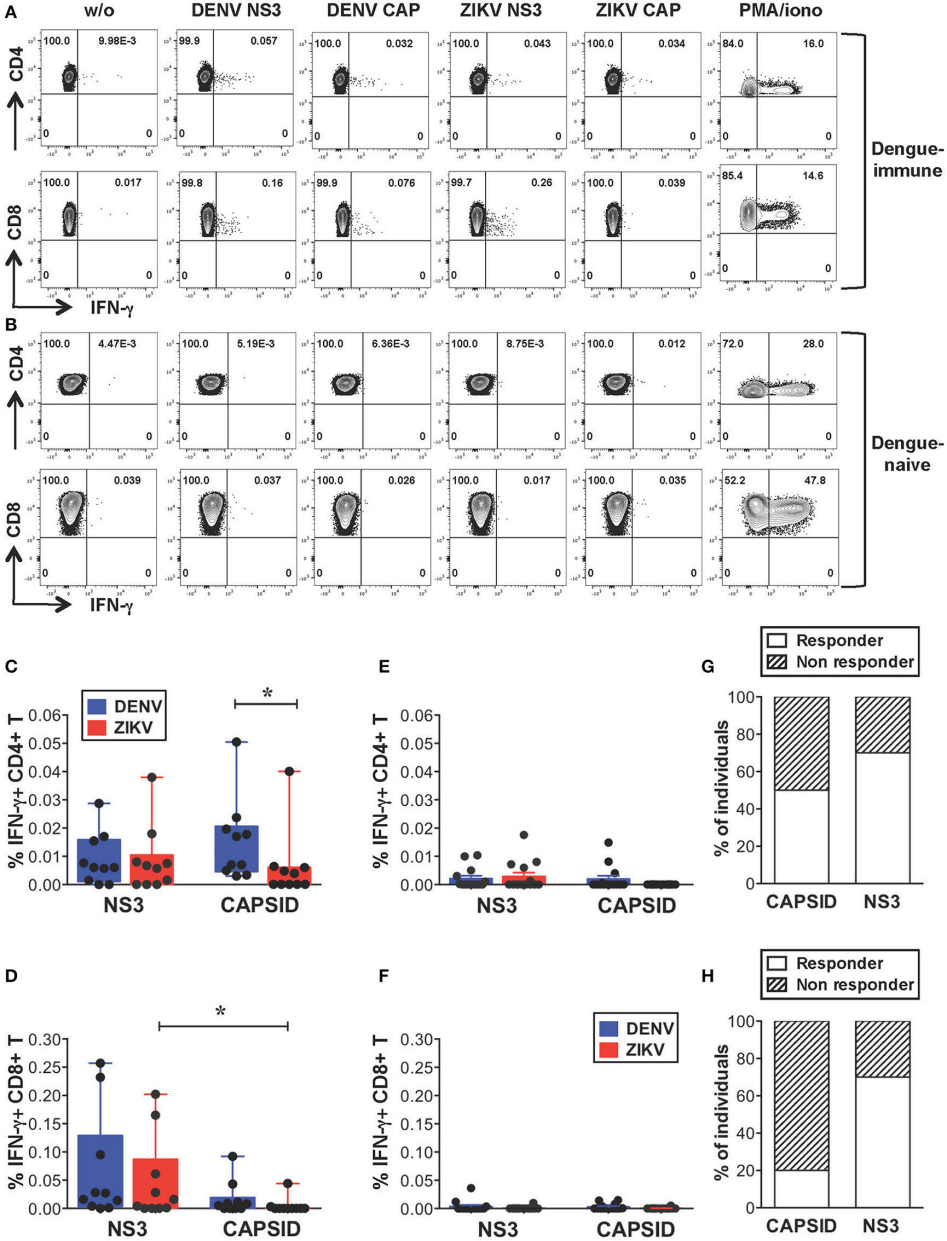
Cross-reactive T cell recognition *ex vivo* of ZIKV peptides in dengue-immune individuals. **(A–F)** *Ex vivo* T cell recognition of DENV and ZIKV NS3 and capsid proteins in dengue-immune and dengue-naïve subjects as assessed by intracellular cytokine staining (ICS) for IFN-γ production after a 5-h stimulation of PBMCs with or without overlapping 15-mer peptides spanning DENV and ZIKV NS3 and capsid proteins. **(A,B)** Dot plots depicting IFN-γ production in CD4+ and CD8+ T cells from one representative dengue-immune **(A)** and dengue-naïve **(B)** donor are shown. Plots are gated on live, CD3+, CD4+, or CD8+ T cells (top or bottom panels, respectively). **(C–F)** CD4+ and CD8+ T cell recognition based on IFN-γ production in the presence of DENV and ZIKV peptides as shown in a/b is summarized for dengue-immune individuals **(C**: CD4+; **D**: CD8+, *n* = 10) and for healthy donors (**E**: CD4+; **F**: CD8+; *n* = 14). Results are expressed as percentage of cytokine positive cells relative to total CD4+ or CD8+ T cells. Statistics are calculated using the non-parametric two-tailed Mann-Whitney test. **(G,H)** The percentage of dengue-immune individuals that display a CD4+ **(G)** and CD8+ **(H)** T cell response to ZIKV capsid and NS3 is summarized.

We first investigated the extent of cross-reactive T cell recognition of ZIKV antigens by dengue-specific memory T cells and asked whether cross-reactivity could be detected in both CD4+ and CD8+ T cell compartments. Due to cell number limitations of our samples we focused our study on the structural protein capsid and the nonstructural protein NS3, which represent the main targets of the CD4+ (capsid, NS3) and CD8+ (NS3) T cell response to DENV (33–35). For these analyses we selected subjects with dengue-resolved infections, no prior exposure to ZIKV and a detectable *ex vivo* IFN-γ T cell response to DENV NS3 and/or capsid proteins (indicated as “dengue-immune”). Healthy donors with no prior exposure to DENV or ZIKV were also analyzed (indicated as “dengue-naive”). Details of subjects are indicated in Supplementary Tables SIA,B. Peripheral blood mononuclear cells (PBMCs) from dengue-immune or dengue-naive donors were stimulated directly *ex vivo* for 5 h with or without 15-mer peptides overlapping by 10 aminoacids and spanning the DENV or ZIKV capsid and NS3 protein sequences. Capsid and NS3 protein sequences are shown in Supplementary Figure S1. Following stimulation, IFN-γ production was measured by intracellular cytokine staining (ICS) and flow cytometry. Dot plots are shown for one representative dengue-immune and dengue-naive donor (Figures 1A,B) and results for all donors are summarized for CD4+ and CD8+ T cells (Figures 1C,D, *n* = 10, and Figures 1E,F, *n* = 14). Our data show that, despite the overall low levels of IFN-γ production in T cells of dengue-immune individuals, CD4+ and CD8+ T cell recognition of ZIKV NS3 protein was present and comparable to that of DENV NS3 (Figures 1C,D). In line with previous studies (33), DENV capsid elicited IFN-γ responses mainly in CD4+ T cells and to a minor extent in CD8+ T cells of dengue-immune donors (Figures 1C,D). Accordingly, cross-reactive recognition of ZIKV capsid was detected only for the CD4+ T cell subset albeit at significantly lower levels compared to its DENV counterpart (Figure 1C). No or low recognition of DENV/ZIKV NS3 and capsid proteins was observed in dengue-naive donors (Figures 1E,F). Serology analysis of convalescent plasma samples that were available for *n* = 10 dengue-immune individuals show lack of neutralizing activity toward ZIKV, as assessed by a standard neutralization assay (36), suggesting that a previous asymptomatic exposure to ZIKV is unlikely (Supplementary Table SIA and Supplementary Methods). Plasma samples from *n* = 3 subjects however show ZIKV NS1 binding activity as assessed by the EUROIMMUN ZIKV IgG assay (Supplementary Table SIA), which is in line with previous studies reporting the cross-reactive recognition of ZIKV proteins by DENV antibodies (10).

In summary, our data show that CD4+ and CD8+ T cells from 70% of individuals with prior dengue immunity are able to recognize peptides from ZIKV NS3 protein (Figures 1G,H). In contrast, peptides from ZIKV capsid protein elicit a significantly lower response compared to the DENV counterpart in 50% of subjects analyzed (Figure 1G). Our data suggest that in dengue-immune individuals cross-reactivity toward ZIKV NS3 protein is robust while that directed against capsid is low or absent.

### DENV/ZIKV cross-reactivity after *in vitro* T cell expansion and identification of cross-reactive epitopes

**Figure 2:**
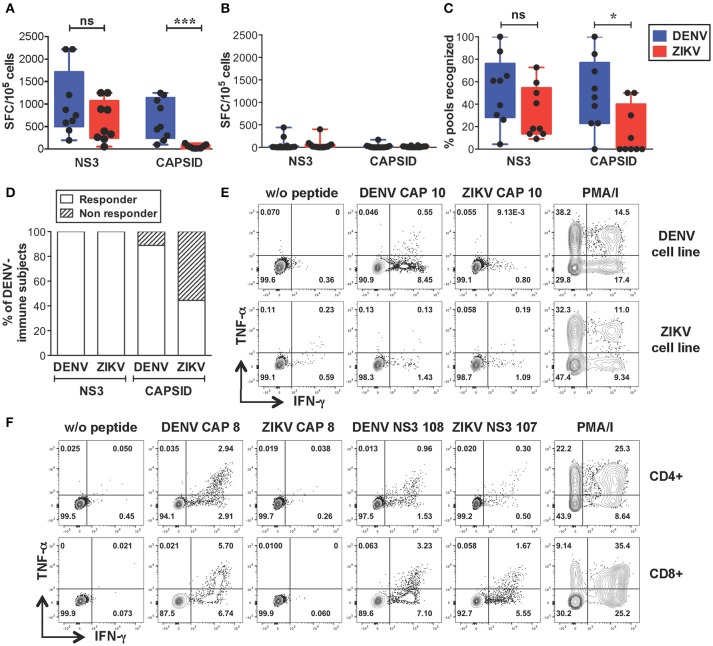
Strong cross-reactive T cell recognition *in vitro* of ZIKV NS3 protein in DENV-immune individuals. **(A,B)** T cells from dengue-immune (**A**, *n* = 9) and dengue-naïve subjects (**B**, *n* = 14) were expanded *in vitro* for 10 days with NS3 and capsid peptides from either DENV or ZIKV. T cell recognition of DENV and ZIKV NS3 and capsid peptides was assessed by IFN-γ ELISPOT. Results are expressed as Spot Forming cells (SFC) relative to 10^5^ cells. Values indicate the sum of responses detected for each peptide pool within a single protein. **(C)** Percentage of peptide pools from DENV and ZIKV proteins that elicit a response in dengue-immune individuals from a, where the total number of peptide pools for DENV NS3, ZIKV NS3, DENV capsid, ZIKV capsid is *n* = 23, 22, 13, and 10, respectively. **(D)** Percentage of dengue-immune subjects (*n* = 9) that respond to at least one DENV and ZIKV NS3 and capsid peptide pool. **(E,F)** Production of IFN-γ and TNF-α by DENV and ZIKV T cell lines upon stimulation with or without single DENV or ZIKV peptides or with PMA/ionomycin, as assessed by ICS. Results are from two representative patients. Plots are gated on live CD4+ (**E,F**, top row) or CD8+ T cells (**F**, bottom row). Statistics are calculated using the non-parametric two-tailed Mann-Whitney test.

We next sought to identify the sequences of cross-reactive T cell epitopes and study the characteristics and functionality of cross-reactive T cells. We selected dengue-immune (*n* = 9) and dengue-naive (*n* = 10) individuals that expressed HLA-A^*^11:01 and/or HLA-A24:02, the two most expressed HLA-A molecules in the Singapore dengue patient cohort (unpublished data). An additional 4 dengue-naive donors with unknown HLA type were included in the analysis (subject details in Supplementary Tables SIIA,B). PBMCs were expanded *in vitro* with overlapping peptides spanning the DENV or ZIKV capsid/NS3 protein sequences resulting in DENV or ZIKV T cell lines, respectively. T cell lines were tested by IFN-γ ELISPOT for recognition of the corresponding peptides, as described previously (33). This method allows to increase the *ex vivo* T cell frequencies while maintaining the overall immunodominance of the antigen-specific T cell response (37). In line with the results from the *ex vivo* analysis, our data show that dengue-immune subjects display strong recognition of ZIKV NS3 peptides, which was not significantly lower than that observed for DENV NS3 peptides (Figure 2A). In contrast, recognition of ZIKV capsid was of lower magnitude compared to that of DENV capsid (Figure 2A), while dengue-naive subjects display no/low recognition of DENV or ZIKV peptides (Figure 2B). The lack of a detectable T cell response toward DENV or ZIKV peptides in dengue-naive donors further confirms that this methodology is unable to elicit a primary T cell response *in vitro*. The breadth of ZIKV NS3 recognition was broad and similar to that of DENV NS3, while it was significantly narrower for ZIKV capsid (Figure 2C). Notably, recognition of ZIKV NS3 could be detected consistently in 100% of individuals tested (*n* = 9/9), while that of ZIKV capsid protein was present only in 44% of individuals (*n* = 4/9, Figure 2D). As observed for the dengue-immune subjects in Figure 1 we could exclude prior exposure to ZIKV by confirming the absence of ZIKV neutralizing antibodies in the corresponding convalescent plasma samples (Supplementary Table SIIA). Plasma from 3 of these subjects however displayed ZIKV NS1 binding antibodies, highlighting the cross-reactive nature of DENV NS1-reactive IgG (Supplementary Table SIIA).

Cross-reactive T cell recognition between DENV and ZIKV was further confirmed at the single peptide level by flow cytometry. Peptides that had elicited a positive response in the ELISPOT assays were deconvoluted based on the presence of each individual peptide in two distinct pools. The peptide of interest was then used to briefly stimulate the T cell lines for 5 h and ICS was performed for detection of IFN-γ, TNF-α, and IL-2 production. Cross-reactive T cells were identified in both the CD4+ and CD8+ T cell compartments and were mainly contained within the NS3 protein (Table 1 and Supplementary Figure S1). Recognition of 5 distinct peptides was confirmed within the DENV capsid protein, 4 of which were CD4+ T cell epitopes (CAP 8, 10, 14, 15) and 1 also contained a CD8+ T cell epitope (CAP 8). Except for CAP 10, which was cross-reactive between DENV and ZIKV (Figure 2E and Table 1, shaded in dark gray), all other epitopes in the capsid protein were recognized only in their DENV variant (Figure 2F and Table 1). Thirteen distinct T cell epitopes were confirmed within the DENV NS3 protein. Of these, 8 were cross-reactive with ZIKV (Figure 2F and Table 1, shaded in dark gray) while 5 where specific for DENV. In summary, out of 18 distinct DENV T cell peptides that were confirmed by flow cytometry, 9 were also recognized in their ZIKV variant and 9 were recognized only in their DENV variant. Our data demonstrates broad CD4+ and CD8+ T cell recognition of ZIKV NS3 protein, and to a lesser extent of ZIKV capsid protein, in individuals with prior immunity to DENV.

**Table 1:** Cross-reactive DENV/ZIKV and non cross-reactive DENV-specific T cell epitopes identified in this study.

**Peptide ID**		**Amino acid sequence**		**Positive ICS response (subset)**		N^°^(ID) of responding subjects^**^	**Source or Reference (HLA restriction^#^, epitope^∧^)**
**DENV**	**ZIKV**	**DENV**	**ZIKV**	DENV peptide^*^	ZIKV peptide^*^		
CAP 8 (36–50)	CAP 8 (36–50)	GMLQGRGPLKLFMAL	GLLLGHGPIRMVLAI	Yes (CD4)	No	1 (#13)	**This study**^1^
CAP 8 (36–50)	CAP 8 (36–50)	GMLQGRGPLKLFMAL	GLLLGHGPIRMVLAI	Yes (CD8)	No	1 (#13)	**This study**^2^
CAP 10 (46–60)	CAP 10 (46–60)	LFMALVAFLRFLTIP	MVLAILAFLRFTAIK	Yes (CD4)	Yes/low (CD4)	3 (#14, #15, #20)	Rivino JVI 2013
CAP 14 (66–80)	CAP 14 (66–80)	LKRWGTIKKSKAINV	INRWGSVGKKEAMEI	Yes (CD4)	No	1 (#14)	Simmons JVI 2005
CAP 15 (71–85)	CAP 15 (71–85)	TIKKSKAINVLRGFR	SVGKKEAMEIIKKFK	Yes (CD4)	No	1 (#20)	Rivino JVI 2013
NS3 25 (121–135)	NS3 24 (116–130)	GTIGAVSLDFSPGTS	DIGAVALDYPAGTSG	Yes (CD4)	No	1 (#20)	Simmons JVI 2005 (AVSLDFSPGTSGSPI)
NS3 44 (216–230)	NS3 43 (211–225)	LRTLILAPTRVVAAE	LRTVILAPTRVVAAE	Yes (CD4)	Yes (CD4)	1 (#13)	**This study**
NS3 56 (276–290)	NS3 55 (271–285)	PNYNLIIMDEAHFTD	PNYNLYIMDEAHFTD	Yes (CD4)	Yes (CD4)	1 (#18)	Simmons JVI 2005, Rivino JVI 2013
NS3 57 (281–295)	NS3 56 (276–290)	IIMDEAHFTDPASIA	YIMDEAHFTDPSSIA	Yes (CD4)	Yes (CD4)	1 (#18)	Simmons JVI 2005 (LIIMDEAHFTDPASI)
NS3 63 (311–325)	NS3 62 (306–320)	GIFMTATPPGSRDPF	AIFMTATPPGTRDAF	Yes (CD4)	Yes (CD4)	1 (#21)	Weiskopf 2016 (AAGIFMTATPPGSRD)
NS3 77 (381–395)	NS3 76 (376–390)	KVIQLSRKTFDSEYV	RVIQLSRKTFETEFQ	Yes (CD4)	ND	1 (#9)	Weiskopf 2016 (DRB1^*^12:02, KKVIQLSRKTFDSEY)
NS3 108 (536–550)	NS3 107 (531–545)	LMRRGDLPVWLAYRV	KRGDLPVWLAYQVAS	Yes (CD8)	Yes (CD8)	2 (#13, #14)	This study (Cw^*^04:03, RRGDLPVWL)^3^
NS3 108 (536–550)	NS3 107 (531–545)	LMRRGDLPVWLAYRV	KRGDLPVWLAYQVAS	Yes (CD4)	Yes (CD4)	2 (#13, #14)	**This study**
NS3 109 (541–555)	NS3-108 (536–560)	DLPVWLAYRVAAEGI	PVWLAYQVASAGITY	Yes (CD8)	No	1 (#14)	Chang Eur J Immunol 2013 (B^*^5502, LPVWLAYRV)
NS3 111 (555–565)	NS3 110 (546–560)	AAEGINYADRRWCFD	AGITYTDRRWCFDGT	ND	Yes (CD4)	1 (#13)	**This study**
NS3 112 (556–570)	NS3 110 (546–560)	NYADRRWCFDGVKNN	AGITYTDRRWCFDGT	Yes (CD8)	Yes (CD8)	2 (#13, #15)	Mongolsapaya J Immunol 2006 (A^*^24, NYADRRWCF)
NS3 120 (596–610)	NS3 119 (591–605)	LDARIYSDPLALKEF	ARVCSDHAALKSFKE	Yes (CD4)	No	1 (#16)	**This study**
NS3 120 (596–610)	NS3 119 (591–605)	LDARIYSDPLALKEF	ARVCSDHAALKSFKE	Yes (CD8∧)	No	1 (#16)	Rivino JVI 2013 (A^*^24:02); Weiskopf J Immunol 2011 (A^*^11:01, RIYSDPLALK)

*Cross-reactive peptides are shaded in dark gray*.

* *Recognition was observed in either the DENV, the ZIKV cell lines or in both*.

** *Total subjects tested by ICS n = 8*.

# *HLA restriction and minimal epitope are shown in bold when they were identified in this study*.

∧ *If different from the 15-mer. The following epitopes were previously reported but the HLA restriction does not match that of our patient*:

1 Weiskopf 2016 (DRB1^*^15:02, LQGRGPLKLFMALVA).

2 Weiskopf 2014 (B^*^08:01, GPLKLFMAL).

3 Piazza Clinical Exp Immunol 2014 (B^*^27:01, MRRGDLPVWL).

### Cytokine profiles of cross-reactive T cells upon recognition of DENV vs. ZIKV peptides

**Figure 3:**
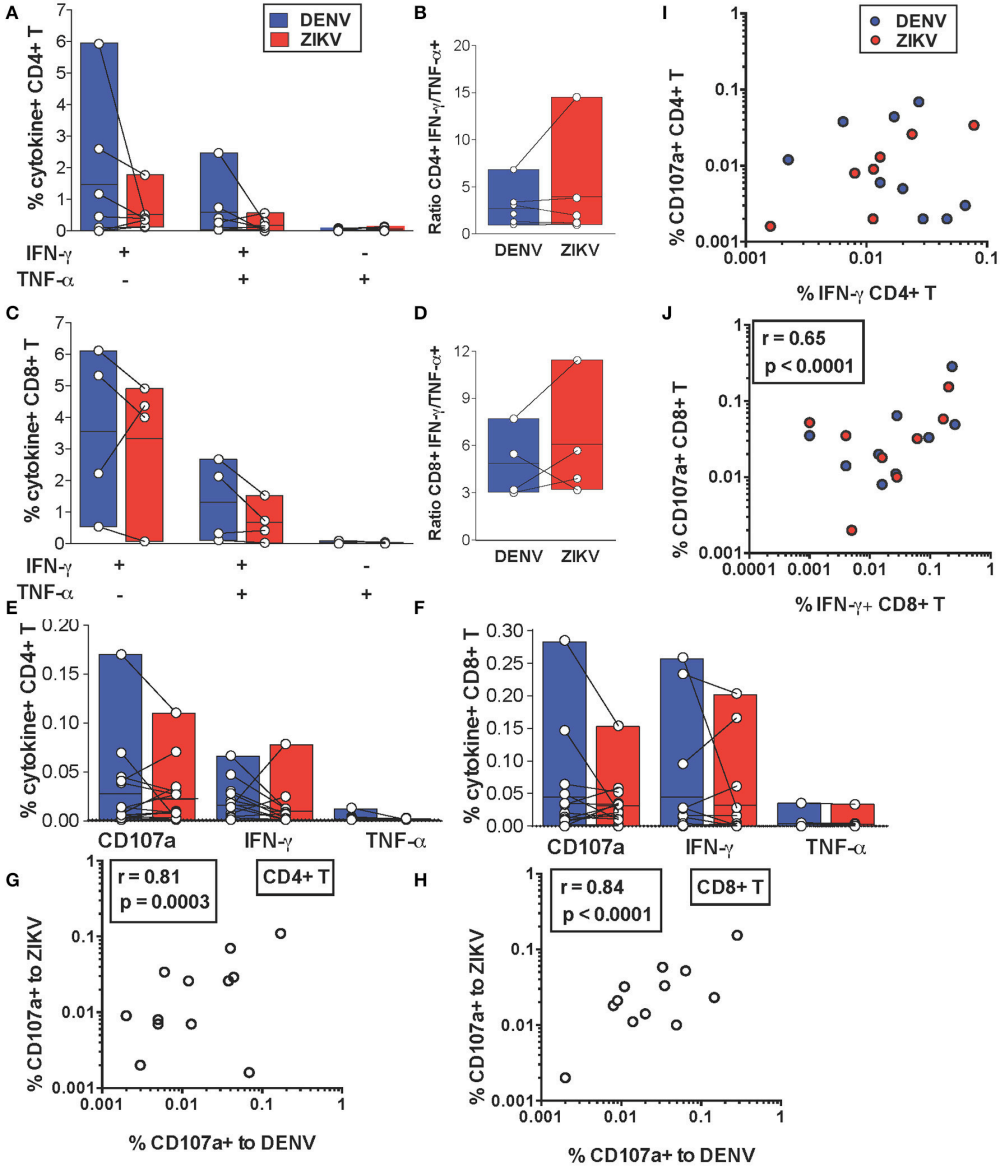
*In vitro* and *ex vivo* cytokine production by cross-reactive T cells upon stimulation with DENV or ZIKV peptides. **(A–D)** IFN-γ and TNF-α production after DENV or ZIKV stimulation is assessed by ICS in CD4+ **(A)** and CD8+ T cell lines **(C)** in dengue-immune subjects (*n* = 7) and is shown as percentages of cytokine+ cells in CD4+ or CD8+ T cells, respectively. Each point indicates a single peptide. The ratios of IFN-γ/TNF-α production in CD4+ **(B)** and CD8+ **(D)** T cell lines from **(A)** and **(C)** are shown for each peptide. **(E,F)** Expression of CD107a and production of IFN-γ and TNF-α after DENV or ZIKV stimulation is assessed directly *ex vivo* by ICS in dengue-immune subjects (*n* = 15). Each point indicates a single subject. All differences are not significant based on the non-parametric two-tailed Mann Whitney test. **(G,H)** Pearson correlation of CD107a expression after DENV or ZIKV stimulation in CD4+ **(G)** or CD8+ T cells **(H)**. **(I,J)** Pearson correlation of CD4+ **(I)** and CD8+ **(J)** T cell expression of CD107a and IFN-γ production. Lines inside bars in **(A-F)** indicate mean.

We next addressed whether cross-reactive T cell recognition of ZIKV peptides is characterized by cytokine profiles that resemble those elicited by the DENV counterpart or if this leads to skewing of T cell functionality toward production of pro-inflammatory cytokines. We compared production of IFN-γ and TNF-α by cross-reactive T cell lines upon recognition of DENV or ZIKV peptides. Across all the identified cross-reactive peptides CD4+ and CD8+ T cell production of IFN-γ and TNF-α did not differ significantly upon recognition of the DENV peptide compared to its ZIKV variant (Figures 3A,C). Accordingly, the IFN-γ/TNF-α T cell ratios were also not significantly different for DENV and ZIKV peptides (Figures 3B,D). These data show that the cross-reactive T cell lines analyzed are not skewed toward production of pro-inflammatory cytokines such as TNF-α, which are believed to potentially play a detrimental role during heterologous T cell immunity.

We further investigated whether cytokine production upon DENV vs. ZIKV peptide stimulation may differ in T cells directly *ex vivo*. IFN-γ and TNF-α production as well as expression of CD107a, an indirect marker of degranulation, were determined by ICS after a brief 5 h stimulation of cells with DENV and ZIKV peptides directly *ex vivo*. Our data show that for both CD4+ and CD8+ T cells CD107a expression and IFN-γ production in cross-reactive T cells was not significantly different upon encounter of DENV and ZIKV capsid and NS3 peptides (Figure 3E) or NS3 peptides (Figure 3F), respectively. Production of TNF-α was largely absent or low in all conditions. In particular, there was a strong direct correlation between CD107a expressed after stimulation with DENV or ZIKV peptide variants, suggesting that cross-reactive T cells may display similar anti-viral effector functions upon encounter of these antigens *in vivo* (Figures 3G,H). There was a direct correlation at the single cell level of CD107a expression and IFN-γ production after stimulation with DENV or ZIKV peptides in CD8+ but not in CD4+ T cells (Figures 3I,J).

In summary, our data show similar production of anti-viral effector mediators in CD4+ and CD8+ T cells upon stimulation with DENV or ZIKV peptides *ex vivo* or after *in vitro* expansion.

### CD8+ T cells specific for DENV NS3 108 display anti-viral effector functions and lytic activity toward ZIKV-infected cells

**Figure 4:**
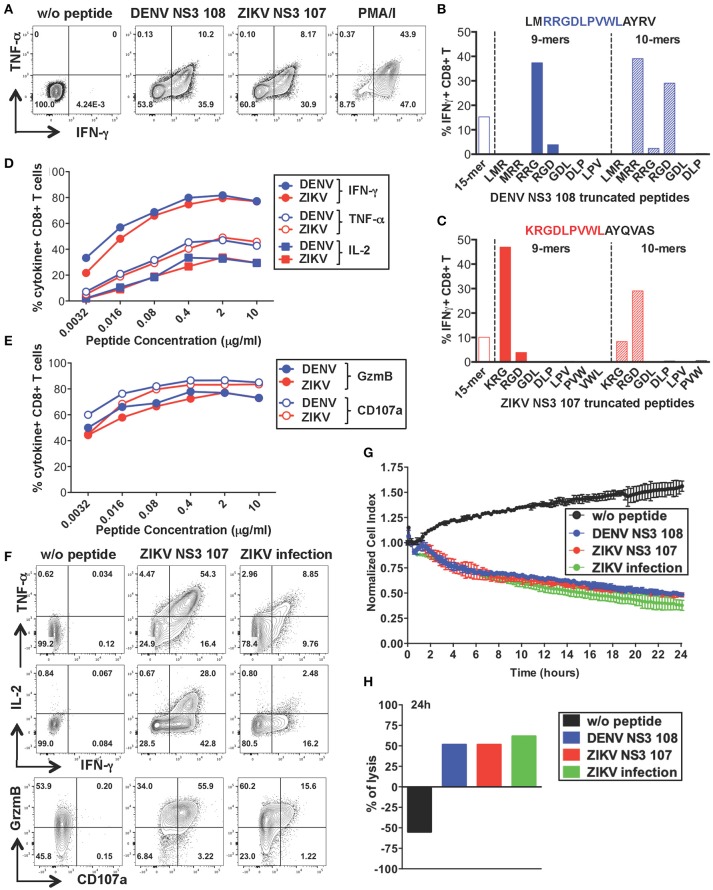
Anti-viral capacity of cross-reactive CD8+ T cells targeting DENV NS3 108. **(A)** Production of IFN-γ and TNF-α after restimulation of DENV T cell lines in the presence or absence of DENV NS3 108 and ZIKV NS3 107 peptides or with PMA/ionomycin. Plots are gated on live, CD8+ T cells. **(B,C)** Characterization of the minimal CD8+ T cell epitope from DENV NS3 108 **(B)** and ZIKV NS3 107 **(C)** capable of inducing optimal IFN-γ production by CD8+ T cell lines. The optimal minimal epitope is highlighted in blue or red. **(D,E)** Production of effector cytokines **(D)** or cytolytic mediators **(E)** by the CD8+ T cell line after 5 h co-culture with WGP 48 cells pulsed with serial dilutions of the 9-mers DENV NS3 108 RRG or ZIKV NS3 107 KRG, as assessed by ICS. **(F)** Production of IFN-γ, TNF-α, granzyme B and CD107a by the CD8+ T cell line upon overnight co-culture with WGP 48 cells that were pulsed with or without ZIKV NS3 107 or were previously infected with ZIKV (strain MR766). **(G)** Kinetics of cellular lysis of WGP 48 cells after co-culture with CD8+ T cell line, measured by xCELLigence Real-Time Cell Analysis (RTCA). Cell lysis is measured as normalized cell index over a 24 h period of WGP 48 cells that were either left untreated (w/o peptide), pulsed with DENV or ZIKV peptides or previously infected with ZIKV (strain MR766). SD error bars were calculated on duplicates. One representative experiment is shown. **(H)** Summary from **(G)** of the percentage of WGP 48 lysis in the presence of the T cell line.

To further characterize the anti-viral capacity of cross-reactive T cells we chose to perform more detailed analysis on cross-reactive T cell lines. The CD8+ T cell line specific for DENV NS3 108 identified in Figure 2 was further expanded with the specific peptide for 10 days to obtain the necessary amount of cells. Peptide-specific CD8+ T cells increased considerably in frequency and recognition of DENV NS3 108 peptide or the corresponding ZIKV NS3 107 peptide elicited similar ratios of TNF-α, IFN-γ, and IL-2 (Figure 4A and Supplementary Figure S2A). To identify the number of amino acid substitutions that occur between DENV NS3 108 and ZIKV NS3 107, we characterized the sequence of the minimal epitope capable of inducing an optimal CD8+ T cell response after stimulating the cells with truncated forms of the peptides (Figures 4B,C). Thus, we identified the 9-mers DENV RRGDLPVWL and ZIKV KRGDLPVWL, which differ in one amino acid (R = arginine → K = lysine) as the minimal cross-reactive DENV/ZIKV CD8+ T cell epitopes. To assess the MHC:peptide:T cell receptor (TCR) avidity of the cross-reactive T cells for DENV RRG or ZIKV KRG 9-mers, HLA class I matched EBV-immortalized B cell lines (WGP 48) were pulsed with serial dilutions of DENV RRG and ZIKV KRG. Cells were then washed and co-cultured with the CD8+ T cell line for 5 h and T cell production of IFN-γ, TNF-α, IL-2, granzyme B and CD107a was assessed by ICS (Figures 4D,E). Stimulation with serial dilutions of the DENV and ZIKV 9-mer peptides elicited comparable levels of cytokines and effector mediators, thus suggesting similar MHC:peptide:TCR avidity for the DENV and ZIKV epitope. Identification of the HLA restriction element was performed as described previously (33) (Supplementary Figure S2B)

During viral infection CD8+ T cells mediate viral clearance through recognition and direct killing of virus-infected cells. We thus asked whether the peptide-specific T cell line could recognize and directly lyse ZIKV-infected target cells. WGP 48 cells were infected with ZIKV and co-cultured with the peptide-specific CD8+ T cell line for 16 h after which T cell production of effector cytokines (IL-2, TNF-α, IFN-γ) and cytolytic mediators (granzyme B and CD107a) was assessed by ICS. Despite the relatively low levels of infectivity of WGP 48 cells (Supplementary Figure S3A), the CD8+ T cell line displayed strong upregulation of CD107a and granzyme B and production of IL-2, IFN-γ and TNF-α in the presence of ZIKV-infected WGP 48 cells (Figure 4F). Importantly, CD8+ T cells could directly lyse ZIKV-infected cells as assessed by xCELLigence Real-Time Cell Analysis (RTCA). We observed similar cytolytic capacity of the peptide-specific CD8+ T cell line toward WGP 48 target cells that were either left unpulsed, pulsed with DENV 108 or ZIKV 107 or infected with ZIKV (Figures 4G,H). These results demonstrate that ZIKV-infected cells efficiently process and present the NS3 107 epitope in association with HLA-I molecules and they can activate cross-reactive DENV-specific T cells. Our data demonstrate that a peptide-specific CD8+ T cell line from a dengue-immune individual can mediate anti-viral effector function and lytic potential toward ZIKV-infected target cells.

### CD4+ T cells specific for DENV NS3 57 display anti-viral effector potential toward ZIKV-infected cells in the absence of cytolytic activity

**Figure 5:**
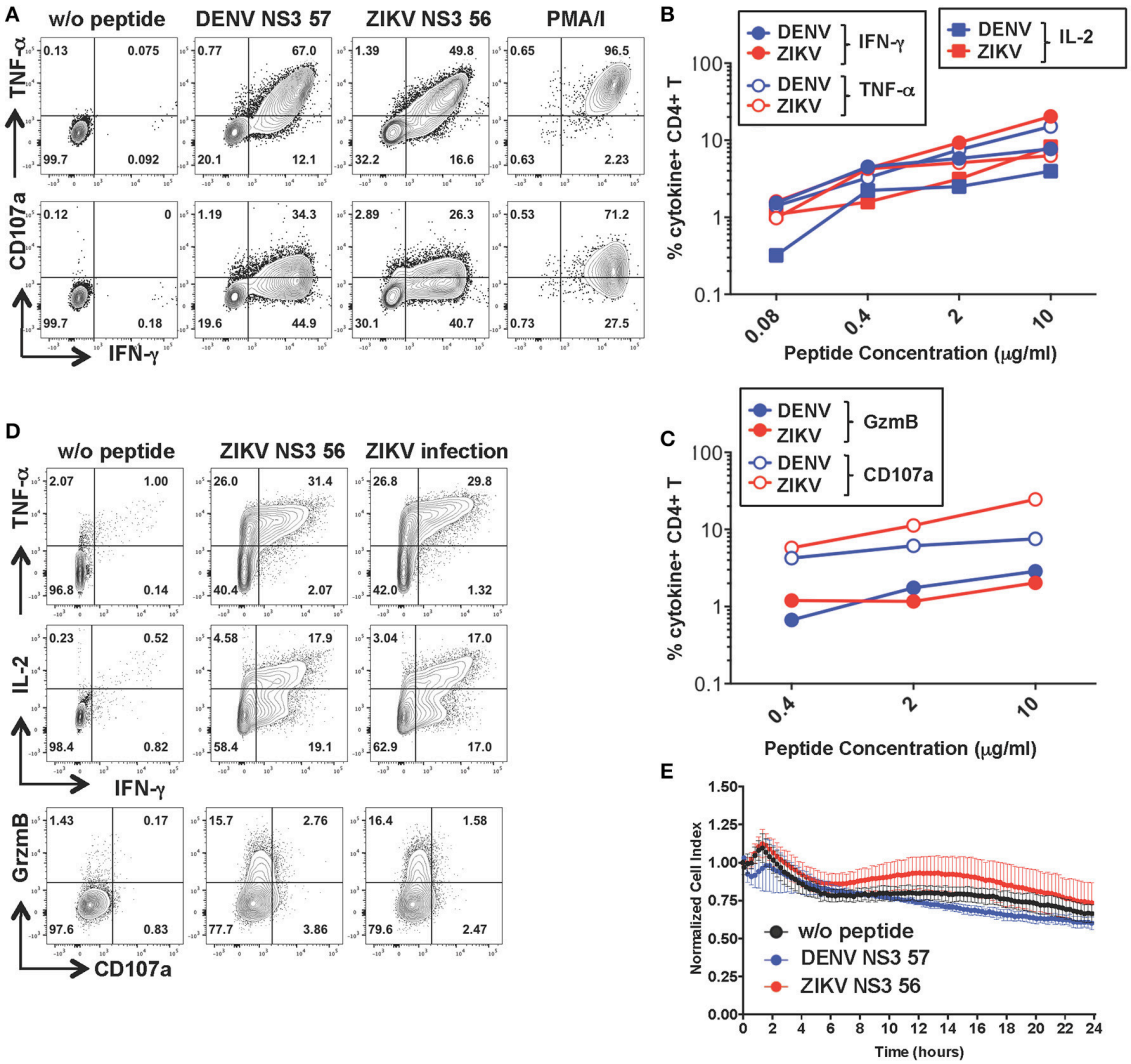
Anti-viral capacity of cross-reactive CD4+ T cells targeting DENV NS3 57. **(A)** Production of IFN-γ, TNF-α, and CD107a after stimulation of CD4+ T cell lines in the presence or absence of DENV NS3 57 and ZIKV NS3 56 peptides or with PMA/ionomycin. Plots are gated on live, CD4+ T cells. **(B,C)** Production of effector cytokines **(B)** or cytolytic mediators **(C)** of the CD4+ T cell line after co-culture with LKC cells pulsed with serial dilutions of DENV NS3 57 or ZIKV NS3 56 peptides, as assessed by ICS. **(D)** Production of IL-2, IFN-γ, TNF-α, granzyme B, and CD107a by the CD4+ T cell line upon overnight co-culture with LKC cells that were pulsed with or without ZIKV NS3 56 or were previously infected with ZIKV (strain MR766). **(E)** Kinetics of cellular lysis of LKC target cells in the presence of the CD4+ T cell line, measured by RTCA. Cell lysis is measured as normalized cell index over a 24 h period in LKC cells that were either left untreated (w/o peptide) or pulsed with DENV or ZIKV peptides. SD error bars are calculated on duplicates. One representative experiment is shown. The peptide sequences of DENV NS3 57 and ZIKA NS3 56 are IIMDEAHFTDPASIA and YIMDEAHFTDPSSIA, respectively.

To investigate the functionality of a cross-reactive CD4+ T cell line specific for DENV NS3 57 we restimulated the cell line from Figure 2 for 10 days with the relevant peptide and obtained a cell line with increased frequencies of peptide-specific cells (Figure 5A). The MHC:peptide:TCR avidity of the CD4+ T cell line for the 15-mer peptides was assessed by using HLA class II matched cells (LKC) pulsed with serial dilutions of the DENV or ZIKV 15-mer peptide (Figures 5B,C; identification of HLA class II matched EBV-immortalized B cell lines is shown in Supplementary Figure S4). The MHC:peptide:TCR avidity of the CD4+ T cell line toward ZIKV NS3 56 was not inferior to that of the corresponding DENV NS3 57 peptide (Figures 5B,C). Similar to what was observed for the CD8+ T cell line, the CD4+ T cell line could recognize ZIKV-infected LKC target cells and exert anti-viral effector functions by producing IL-2, IFN-γ, TNF-α, and granzyme B. However, in these conditions CD107a expression could not be detected either upon co-culture of T cells with ZIKV-infected cells or with the relevant peptide (Figure 5D and Supplementary Figure S3B). Accordingly, despite efficient recognition of LKC cells pulsed with DENV NS3 57 or ZIKV NS3 56 and production of anti-viral effector mediators, the CD4+ T cell line lacked direct cytotoxic capacity (Figure 5E), as assessed by RTCA. Similar findings emerge from the analysis of a cross-reactive CD4+ T cell line specific for DENV NS3 63. The MHC:peptide:TCR avidity of the CD4+ T cell line for DENV NS3 63 and ZIKV NS3 62 peptides was comparable and encounter of these peptides elicited production of similar amounts of anti-viral mediators, in the absence of cytolytic capacity (Supplementary Figures S5A–D). Moreover, the CD4+ NS3 63 cell line could recognize ZIKV-infected autologous EBV cell lines (Supplementary Figure S5C). Further studies are addressing these findings, which may indicate a distinct anti-viral role of DENV-specific CD4+ T cells that does not involve direct killing.

In conclusion, our data demonstrate that individuals with prior immunity to DENV display cross-reactive CD4+ and CD8+ memory T cells that can exert anti-viral effector functions toward ZIKV-infected cells *in vitro* by producing anti-viral mediators (IFN-γ, TNF-α, IL-2, and granzyme B). In addition, cross-reactive DENV-specific CD8+ T cells display direct cytolytic ability toward ZIKV-infected cells. These findings support recent studies in mice and suggest that in humans pre-existing T cell immunity toward DENV may provide a similar immune-protective role toward ZIKV infection.

## Discussion

Heterologous T cell immunity has been investigated mostly in mice infected with various combinations of viruses (38) and was shown to lead to more rapid clearance and increased survival in response to lethal doses of viruses (e.g., lymphocytic choriomeningitis virus, vaccinia virus, pichinde virus). In these models protection is mediated by IFN-γ released by cross-reactive CD4+ and CD8+ T cells (38, 39) or CD8+ T cell-mediated cytotoxicity (17). Mouse models for secondary DENV infection have demonstrated similar protective effects mediated by pre-existing memory T cells specific for a different DENV serotype (28). More recent mouse studies of ZIKV infection of dengue-immune animals are in line with these results (30, 31). However, the scenario is more complex in humans where cross-reactive T cell recognition is influenced by factors such as the unknown and unique antigenic histories of each individual as well as genetic differences in HLA and TCR repertoires which will influence the outcome (40). Previous studies have addressed T cell cross-reactivity in human cells by analyzing T cells targeting single epitope specificities (20–24). In secondary DENV infection of HLA-A^*^11:01+ patients, disease severity correlates with a more vigorous but suboptimal T cell response toward a specific NS3 peptide leading to altered cytokine production, as assessed using peptide-HLA tetramers (18, 25). These studies are highly informative of the molecular mechanisms underlying T cell cross-reactivity for specific T cell epitopes. However, this may not always reflect what occurs for other T cell specificities or at the level of the total T cell response, which is composed of the sum of all the specificities restricted to the 6 HLA-A, B and C molecules. For dengue infection the breadth of the T cell response is particularly broad and there is a lack of strong immunodominant epitopes that account for the bulk of the response (33, 34). Interestingly, when total T cells targeting mixtures of peptides spanning the whole proteome were analyzed, T cell cytokine skewing was not observed (26). To understand the impact of DENV/ZIKV cross-reactivity on the T cell response we sought to define T cell cross-reactivity toward peptides spanning the entire sequences of capsid and NS3, 2 immunodominant proteins in the context of DENV infection. Our data suggests that *ex vivo* T cell cross-reactivity toward ZIKV NS3 peptides is robust, whereas that targeting capsid is low or absent. As seen in other studies, DENV-specific T cell frequencies *ex vivo* in dengue-resolved infections are low, particularly for CD4+ T cells. We thus validated our findings after a 10 days T cell expansion and performed more detailed analysis on these T cell lines. For the *in vitro* study we chose a group of dengue-immune individuals that were HLA-A^*^11:01+ and HLA-A^*^24:02+ to analyze a more homogeneous group of individuals. Extensive cross-reactive recognition of ZIKV NS3 and lack of recognition of ZIKV capsid is confirmed in T cell lines. The higher sequence homology between DENV and ZIKV NS3 proteins compared to that of capsid proteins could account for these differences (67 vs. 44%, respectively). The smaller size of capsid compared to NS3 could also contribute to these findings (114 vs. 612 amino acids, respectively, Supplementary Figure S1). By performing ICS on the T cell lines we identify *n* = 18 distinct DENV T cell epitopes (capsid *n* = 5; NS3 *n* = 13). Of these, *n* = 1/5 and *n* = 8/13 are cross-reactive to ZIKV within capsid and NS3, respectively. Overall, cytokine profiles did not differ significantly upon recognition of DENV or ZIKV peptides both *ex vivo* and *in vitro* (Figure 3). Interestingly, we observe enhanced IFN-γ responses toward the HLA-A^*^24:02-restricted ZIKV peptide NS3 110 compared to the DENV NS3 112 variant in subject #13 but not in subject #15 (Figure 3C, 2 lower values for DENV, first column on the left). This result further demonstrates how the cross-reactive T cell response to a specific epitope may vary between individuals, possibly because of the distinct TCR repertoires of the T cell lines in the two individuals.

CD4+ and CD8+ T cell expression of CD107a *ex vivo* was not significantly different after encounter of DENV or ZIKV peptides. CD107a (LAMP-1) is a lysosomal associated membrane glycoprotein, which is exposed on the surface of cells upon T cell degranulation of lytic granules. In CD8+ T cells CD107a tends to be co-expressed with IFN-γ at the single cell level, while this does not occur for CD4+ T cells (Figures 3I,J). These results could be explained by recent findings showing that cytotoxic functions in human CD4+ T cells are largely restricted to T_EMRA_ cells compared to T_EM_ and T_CM_ cells, whereas IFN-γ production may be higher in the T_EM_ compartment (41). CD107a expression is maintained in the NS3 108 CD8+ T cell line after *in vitro* expansion and correlates with efficient T cell killing (Figures 4F–H). In contrast, we observe loss of CD107a expression after overnight co-culture of CD4+ T cell lines with target cells pulsed with peptide or infected with ZIKV. Accordingly, loss of CD107a was associated with lack of lytic activity toward both DENV and ZIKV peptide-pulsed cells (Figures 5D,E and Supplementary Figures S5C,D). Current studies are addressing the heterogeneity of the CD4+ T cell response and the lytic capacity of cross-reactive CD4+ and CD8+ T cell clones.

In summary, this study shows that memory T cells from dengue-immune, zika-naive individuals can recognize ZIKV NS3 peptides directly *ex vivo* and after *in vitro* expansion. Cross-reactive recognition leads to similar production of anti-viral effector mediators, suggesting that these cells at least *in vitro* are equally effective against ZIKV infection. This data is in line with recent findings from mouse models that demonstrate a protective role of cross-reactive T cells during ZIKV infection of dengue-immune animals. However, our analysis performed on a small number of subjects represents a proof-of-concept study that should be confirmed in a larger number of individuals. Identifying cross-reactive T cell epitopes in the context of HLA molecules that are expressed by individuals living in dengue-endemic regions and defining T cell functionality associated with recognition of DENV vs. ZIKV peptides provides important clues toward our understanding of heterologous T cell immunity between these two viruses.

## Methods

### Subjects

Human peripheral blood samples were obtained from convalescent dengue patients after written informed consent. The study was conducted in accordance with the Declaration of Helsinki and approved by the Singapore National Healthcare Group ethical review board (DSRB 2008/00293 and DSRB 2013/00209). The details of the dengue-immune and dengue-naive individuals recruited for this study are summarized in Supplementary Tables SI–SIII. Dengue diagnosis was confirmed on the basis of either detection of DENV RNA by reverse transcription-PCR (RT-PCR) or of NS1 antigen by enzyme-linked immunosorbent assay (ELISA) (Bio-Rad). Some patients with positive IgM and IgG acute serology (Panbio Dengue Duo Cassette) were also included if they fulfilled the World Health Organization criteria for probable dengue (18). All patients were classified as non-severe dengue based on the 2009 WHO guidelines (Supplementary Tables SI–SIII). Secondary infections were defined as a DENV-specific IgM/IgG ratio of < 1.8 in paired acute and convalescent plasma samples, as assessed with a PanBio IgM and IgG capture ELISA. The presence of ZIKV-reactive antibodies was assessed by NS1 ELISA (EUROIMMUN).

This is an exploratory study aimed at investigating whether T cells from dengue-immune subjects can recognize antigens from ZIKV. Since there is no published information describing the extent of T cell cross-reactivity between DENV and ZIKV in a Singapore patient cohort it was not possible to calculate a priori the number of dengue-immune subjects needed to ensure adequate power to the study.

### Synthetic peptides

Peptides spanning the NS3 and capsid sequence of DENV 2 (D2/SG/05K4155DK1/2005; Singapore isolate) or ZIKV (Zika virus isolate Zika virus/SZ01/2016; Asian genotype) were purchased from Mimotopes (Australia). The peptides consist of 122 (DENV NS3), 21 (DENV Capsid), 121 (ZIKV NS3), and 23 (ZIKV capsid) 15-mers overlapping by 10 amino acids. The purity of the peptides was above 80% and their composition was confirmed by mass spectrometry analysis. All peptides were dissolved in dimethyl sulfoxide (DMSO) at a concentration of 40 mg/ml, and intermediate working dilutions were performed in RPMI supplemented with 200 μ/ml penicillin and 200 μg/ml streptomycin. For the Enzyme-linked immunosorbent spot (ELISPOT) assays peptides were pooled in a matrix so that each individual peptide was present in 2 different pools as described previously (33). Capsid peptides were pooled in a 5-by-5 matrix containing 4, 5, or 6 peptides; NS3 peptides were pooled in a 11-by-12 matrix containing 11 or 12 peptides. For the ICS experiments overlapping peptides were pooled into a single pool for each protein.

### PBMC isolation and T cell lines

Blood samples were collected in EDTA Vacutainer tubes and peripheral blood mononuclear cells (PBMCs) were isolated from peripheral blood by Ficoll gradient purification and cryopreserved. Cells were thawed on the day of the experiment and expanded with the DENV or ZIKV peptide mixtures as follows. Twenty percentage of PBMCs were pulsed with 10 μg/ml of the overlapping DENV or ZIKV peptides for 1 h at 37°C, subsequently washed and co-cultured with the remaining cells in AIM-V media (Gibco) supplemented with 2% AB human serum (Gibco). T cell lines were cultured for 10 days in the presence of 20 U/ml of recombinant IL-2 (R and D systems). T cell lines generated with the DENV or ZIKV overlapping peptides are referred to as DENV and ZIKV cell lines, respectively. For further characterization of peptide-specific T cells (Figures 4, **5**) DENV or ZIKV T cell lines were restimulated for 10 days with irradiated autologous PHA blasts pulsed with the peptide of interest, irradiated allogenic PBMCs, IL-2 (20 μ/ml), IL-7 (10 ng/ml), and IL-15 (10 ng/ml).

### IFN-γ ELISPOT assay

ELISPOT assays for the detection of IFN-γ-producing cells were performed as described previously (33). Equal number of cells from the DENV or ZIKV cell lines were incubated for 16 h with or without NS3 and capsid overlapping peptide pools from DENV or ZIKV, respectively at a concentration of 5 μg/ml. As a positive control, cells were stimulated with phorbol-12-myristate-13-acetate (PMA, 10 ng/ml) and ionomycin (100 ng/ml). Values are considered positive if they were equal or < 5 spots and at least 2 times above the means of the unstimulated control wells. ELISPOT assays on the expanded cell lines were performed once. Positive responses to peptides were then confirmed by intracellular cytokine staining (ICS) after stimulation of cells with the individual peptide.

### Intracellular cytokine staining (ICS)

PBMCs were thawed and stimulated directly *ex vivo* with or without peptides spanning DENV and ZIKV NS3 and capsid peptide pools (1 μg/ml), or with PMA/ionomycin as a positive control for 5 h at 37°C in the presence of Brefeldin A (10 μg/ml, Sigma-Aldrich). Anti CD107a FITC (clone H4A3) antibody was added to the cells at the beginning of the stimulation to assess their degranulation capacity. Similarly, T cells lines were stimulated with or without the peptide of interest (1 μg/ml) with in some cases addition of anti CD107a FITC antibody at the beginning of the stimulation. After stimulation cells were stained with the yellow LIVE/DEAD fixable dead cell stain kit (Invitrogen) and surface stained with anti CD8 PECY7 (clone RPA-T8) and anti CD4 BV 650 (clone RPA-T4) antibodies, then fixed, and permeabilized using the Cytofix/Cytoperm kit (BD Pharmingen). Cells were then stained with anti IFN-γ PE (R&D, clone 25723), anti IL-2 APC (eBioscience, clone MQ1-17H12), and/or anti TNF-α FITC antibodies (clone 6401.1111) and analyzed on a BD LSR II. Antibodies were purchased from BD unless otherwise stated.

### Epstein-barr virus immortalized B lymphocyte cell lines (EBV cells)

EBV cells matched for one or more HLA molecules with the patient were kindly provided by Chan Soh Ha, WHO Immunology Training and Research Centre, NUS, Singapore. Cells were grown and maintained in R10 media which was prepared as follows: RPMI 1640 medium (Gibco) supplemented with 10% heat-inactivated fetal bovine serum (Sigma-Aldrich), 20 mM HEPES, 0.5 mM sodium pyruvate, 100 μ/ml penicillin, 100 γ/ml streptomycin, minimum essential medium (MEM) amino acids with L-glutamine and MEM non-essential amino acids (all purchased from Gibco). Plasmocin (Invivogen) was added to R10 media to prevent mycoplasma infection of cells. Cell lines tested negative for mycoplasma upon introduction into our laboratory and were subsequently maintained in R10 media containing plasmocin.

### ZIKV infection and T cell assays

EBV cell lines were infected with ZIKV strain MR 766 (Uganda; Genbank ID LC002520) for 48 h at a MOI of 10. Cells were washed and staining was performed on a proportion of cells using a mouse 4G2 antibody (kindly provided by Eng Eong Ooi) and a secondary anti-mouse IgG Alexa Fluor 488 antibody (Life Technologies) to determine the efficiency of infection. EBV cell lines that were infected with ZIKV, uninfected or pulsed with the relevant peptide were then incubated with peptide-specific T cell lines at a ratio of 1:1 for 16 h in the presence of anti CD107a FITC (clone H4A3) antibody and Brefeldin A (2 μg/ml). Cells were subsequently stained with a yellow LIVE/DEAD fixable dead cell stain kit and with anti CD3 PerCP-CY5.5 (clone SP34-2), anti CD4 BV 650 (clone RPA-T4), anti CD8 APC CY7 antibodies (clone SK1), fixed and permeabilized, and ICS was performed using anti IFN-γ V450 (clone B27), anti TNF-α PECY7 (clone MAb11), anti IL-2 PE (clone 5344.111), and anti-Granzyme B Alexa Fluor 647 (clone GB11) antibodies. Cells were analyzed on a BD LSR II analyzer. T cell recognition of ZIKV infected cells was performed once.

### xCELLigence real-time cell analysis (RTCA) systems

Microtiter plates (E-plate 16) were coated with anti CD40 antibody (4 μg/ml, clone 5C3) overnight at room temperature. The antibody coating solution was then removed by flicking and the wells were washed twice with PBS, followed by the addition of 50 μl R10 media to each well. Background impedance in the absence of cells was then measured on the RTCA system. EBV cells were pulsed with or without the peptide of interest (10 μg/ml) in R10 media for 1 h at 37°C, followed by 2 washes. Untreated, peptide-pulsed or ZIKV-infected EBV B cells were added to each well at 80,000 cells/100 μl R10 media and the E-plate was left at room temperature for 30 min to facilitate uniform immobilization of EBV B cells on well bottoms. The plate was then placed back into the RTCA instrument and impedance was measured at 5 min interval for 1 h 30 min. Unbound EBV B cells were then removed and wells were washed once with AIM-V media supplemented with 2% AB human serum, followed by the addition of effector T cells. EBV B and antigen-specific T cells were added at a 1:1 ratio, where the ratio was calculated based on the percentage of virus-specific T cells as assessed by ICS. The plate was placed back into the RTCA instrument and impedance was measured at 15 min interval for 24 h. The killing capacity of CD4+ and CD8+ cell lines was confirmed in two independent experiments (one representative experiment is shown in Figures 4G, **5E**).

### Statistical analyses

All statistics were calculated with Graph Pad Prism version 6 by using a non-parametric two-tailed Mann-Whitney test. Differences were considered significant if below 0.05 and are indicated as follows: ^*^p < 0.05, ^**^p < 0.01, ^***^p < 0.001.

## Data availability

The data sets generated and/or analyzed during the current study are available from the corresponding author on reasonable request.

## Author contributions

LR and AB conceived the study and provided funding. LR designed the experiments, analyzed the data, and wrote the manuscript. ML performed the experiments and analyzed the data. EK and HT performed some experiments. DL and YL recruited the subjects and provided clinical expertise for dengue diagnosis. EO supervised and funded the serology experiments and provided expertise for the DENV and ZIKV infection. PM funded the subject recruitment and HLA tissue typing. All authors provided critical review and approved the manuscript.

### Conflict of interest statement

The authors declare that the research was conducted in the absence of any commercial or financial relationships that could be construed as a potential conflict of interest. The reviewer DW declared a past co-authorship with several of the authors ML, PM, LR to the handling Editor.
