# Supplementary material for: Cross-Reactivity and Anti-viral Function of Dengue Capsid and NS3-Specific Memory T Cells Toward Zika Virus
Source: Front Immunol. 2018 Oct 1;9:2225. doi: 10.3389/fimmu.2018.02225 (PMC6174860; doi:10.3389/fimmu.2018.02225)
Supplement: Supplementary file 1 [file Data_Sheet_1.PDF]

A

```
CAPSID                                  20                                 40     CAP 8          CAP 10        60            CAP 14
DENV 2     MNNQRKKAKN TPF-NMLKRE RNRVSTVQQL TKRFSLGMLQ GRGPLKLFMA LVAFLRFLTI PPTAGILKRW 69
ZIKA SZ01  MKNPKKKSGG FRIVNMLKRG VARVSPFGGL -KRLPAGLLL GHGPIRMVLA ILAFLRFTAI KPSLGLINRW 69
ZIKA MR766 MKNPKKKSGG FRIVNMLKRG VARVNPLGGL -KRLPAGLLL GHGPIRMVLA ILAFLRFTAI KPSLGLINRW 69
Consensus  M-N--KK--- ---VNMLKR- --RV-----L TKR---G-L- G-GP-----A --AFLRF--I -P--G---RW

                    80 CAP 15                100                              120
DENV 2     GTIKKSKAIN VLRGFRKEIG RMLNILN--- -RRRRSA--- -GMIIMLIPT VMA 114
ZIKA SZ01  GSVGKKEAME IIKKFKKDLA AMLRIINARK EKKRRGADTN VGIVGLLLTT AMA 122
ZIKA MR766 GSVGKKEAME IIKKFKKDLA AMLRIINARK ERKRRGADTS IGIIGLLLTT AMA 122
Consensus  G---K--A-- ----F-K--- -ML-I-NARK E--RR-ADT- -G----L--T -MA
```

B

```
NS3                                     20                                 40                                 60
DENV 2     VPSPPPMGKA ELEDGAYRIK QKGILGYSQI GAGVYKEGTF HTMWHVTRGA VLMHKGKRIE PSWADVKKDL 70
ZIKA SZ01  VPAPKEVKKG ETTDGVYRVM TRRLLGSTQV GVGVMQEGVF HTMWHVTKGS ALRSGEGRLD PYWGDVKQDL 70
ZIKA MR766 VPAPKEVKKG ETTDGVYRVM TRRLLGSTQV GVGVMQEGVF HTMWHVTKGA ALRSGEGRLD PYWGDVKQDL 70
Consensus  VP-P----K- E--DG-YR-- ----LG--Q- G-GV--EG-F HTMWHVT-G- -L-----R-- P-W-DVK-DL

                    80                                 100                         120 NS3 25               140
DENV 2     ISYGGGWKLE GEWKEGEEVQ VLALEPGKNP RAVQTKPGLF KTNAGTIGAV SLDFSPGTSG SPIIDKKGKV 140
ZIKA SZ01  VSYCGPWKLD AAWDGHSEVQ LLAVPPGERA RNIQTLPGIF KTKDGDIGAV ALDYPAGTSG SPILDKCGRV 140
ZIKA MR766 VSYCGPWKLD AAWDGLSEVQ LLAVPPGERA RNIQTLPGIF KTKDGDIGAV ALDYPAGTSG SPILDKCGRV 140
Consensus  -SY-G-WKL- --W----EVQ -LA--PG--- R--QT-PG-F KT--G-IGAV -LD---GTSG SPI-DK-G-V

                                        160                                180                                200
DENV 2     VGLYGNGVVT RSGAYVSAIA QTEKSIEDNP E-IEDDIFRK RRLTIMDLHP GAGKTKRYLP AIVREAIKRG 209
ZIKA SZ01  IGLYGNGVVI KNGSYVSAIT QGRREEETPV ECFEPSMLKK KQLTVLDLHP GAGKTRRVLP EIVREAIKTR 210
ZIKA MR766 IGLYGNGVVI KNGSYVSAIT QGKREEETPV ECFEPSMLKK KQLTVLDLHP GAGKTRRVLP EIVREAIKKR 210
Consensus  -GLYGNGVV- --G-YVSAI- Q-----E--- EC-E-----K --LT--DLHP GAGKT-R-LP -IVREAIK--

              NS3 44 220                              240                                260              NS3 56 280
DENV 2     LRTLILAPTR VVAAEMEEAL RGLPIRYQTP AIRAEHTGRE IVDLMCHATF TMRLLSPVRV PNYNLIIMDE 279
ZIKA SZ01  LRTVILAPTR VVAAEMEEAL RGLPVRYMTT AVNVTHSGTE IVDLMCHATF TSRLLQPIRV PNYNLYIMDE 280
ZIKA MR766 LRTVILAPTR VVAAEMEEAL RGLPVRYMTT AVNVTHSGTE IVDLMCHATF TSRLLQPIRV PNYNLYIMDE 280
Consensus  LRT-ILAPTR VVAAEMEEAL RGLP-RY-T- A----H-G-E IVDLMCHATF T-RLL-P-RV PNYNL-IMDE

                 NS3 57                 300          NS3 63          320                                340
DENV 2     AHFTDPASIA ARGYISTRVE MGEAAGIFMT ATPPGSRDPF PQSNAPIIDE EREIPERSWN SGHEWVTDFK 349
ZIKA SZ01  AHFTDPSSIA ARGYISTRVE MGEAAAIFMT ATPPGTRDAF PDSNSPIMDT EVEVPERAWS SGFDWVTDHS 350
ZIKA MR766 AHFTDPSSIA ARGYISTRVE MGEAAAIFMT ATPPGTRDAF PDSNSPIMDT EVEVPERAWS SGFDWVTDHS 350
Consensus  AHFTDP-SIA ARGYISTRVE MGEAA-IFMT ATPPG-RD-F P-SN-PI-D- E-E-PER-W- SG--WVTD--

                    360                      380 NS3 77 (Zika:nd)              400                                420
DENV 2     GKTVWFVPSI KAGNDIAACL RKNGKKVIQL SRKTFDSEYV KTRTNDWDFV VTTDISEMGA NFKAERVIDP 419
ZIKA SZ01  GKTVWFVPSV RNGNEIAACL TKAGKRVIQL SRKTFETEFQ KTKHQEWDFV VTTDISEMGA NFKADRVIDS 420
ZIKA MR766 GKTVWFVPSV RNGNEIAACL TKAGKRVIQL SRKTFETEFQ KTKNQEWDFV ITTDISEMGA NFKADRVIDS 420
Consensus  GKTVWFVPS- --GN-IAACL -K-GK-VIQL SRKTF--E-- KT----WDFV -TTDISEMGA NFKA-RVID-

                                        440                                460                                480
DENV 2     RRCMKPVILT DGEERVILAG PMPVTHSSAA QRRGRIGRNP KNENDQYIYM GEPLENDEDC AHWKEAKMLL 489
ZIKA SZ01  RRCLKPVIL- DGE-RVILAG PMPVTHASAA QRRGRIGRNP NKPGDEYLYG GGCAETDEDH AHWLEARMLL 488
ZIKA MR766 RRCLKPVIL- DGE-RVILAG PMPVTHASAA QRRGRIGRNP NKPGDEYMYG GGCAETDEGH AHWLEARMLL 488
Consensus  RRC-KPVILT DGEERVILAG PMPVTH-SAA QRRGRIGRNP ----D-Y-Y- G---E-DE-- AHW-EA-MLL

                    500                                520      NS3 107  NS3 108 540 NS3 109    NS3 112 560
DENV 2     DNINTPEGII PSMFEPEREK VDAIDGEYRL RGEARKTFVD LMRRGDLPVW LAYRVAAEGI NYADRRWCFD 559
ZIKA SZ01  DNIYLQDGLI ASLYRPEADK VAAIEGEFKL KTEQRKTFVE LMKRGDLPVW LAYQVASAGI TYTDRRWCFD 558
ZIKA MR766 DNIYLQDGLI ASLYRPEADK VAAIEGEFKL RTEQRKTFVE LMKRGDLPVW LAYQVASAGI TYTDRRWCFD 558
Consensus  DNI----G-I -S---PE--K V-AI-GE--L --E-RKTFV- LM-RGDLPVW LAY-VA--GI -Y-DRRWCFD

                               580              NS3 120 600
DENV 2     GVKNNQILEE NVEVEIWTKE GERKKLKPRW LDARIYSDPL ALKEFKEFAA GRK-- 612
ZIKA SZ01  GTTNNTIMED SVPAEVWTRH GEKRVLKPRW MDARVCSDHA ALKSFKEFAA GKRGA 613
ZIKA MR766 GTTNNTIMED SVPAEVWTKY GEKRVLKPRW MDARVCSDHA ALKSFKEFAA GKRGA 613
Consensus  G--NN-I-E- -V--E-WT-- GE---LKPRW -DAR--SD-- ALK-FKEFAA G--GA
```

Cross-reactive epitopes   DENV epitopes

Supplementary Figure S1. Alignment of the amino acid sequences of capsid and NS3 proteins from DENV 2 and ZIKV. The amino acid sequences for capsid **(A)** and NS3 **(B)** from DENV 2 (D2/SG/05K4155DK1/2005 strain) and two ZIKV strains (Asian strain SZ01-2016; African strain MR766) are shown. Identical residues are indicated in blue; consensus sequence is indicated below. The cross-reactive or DENV-specific T cell epitopes identified in this study and listed in Table I are indicated here in purple and green, respectively. Peptide libraries were designed based on the indicated DENV 2 and ZIKV SZ01-2016 sequences, while ZIKV MR766 was used for the infection experiments. The sequence of all immunogenic peptides identified in this study was identical for the two ZIKV sequences.

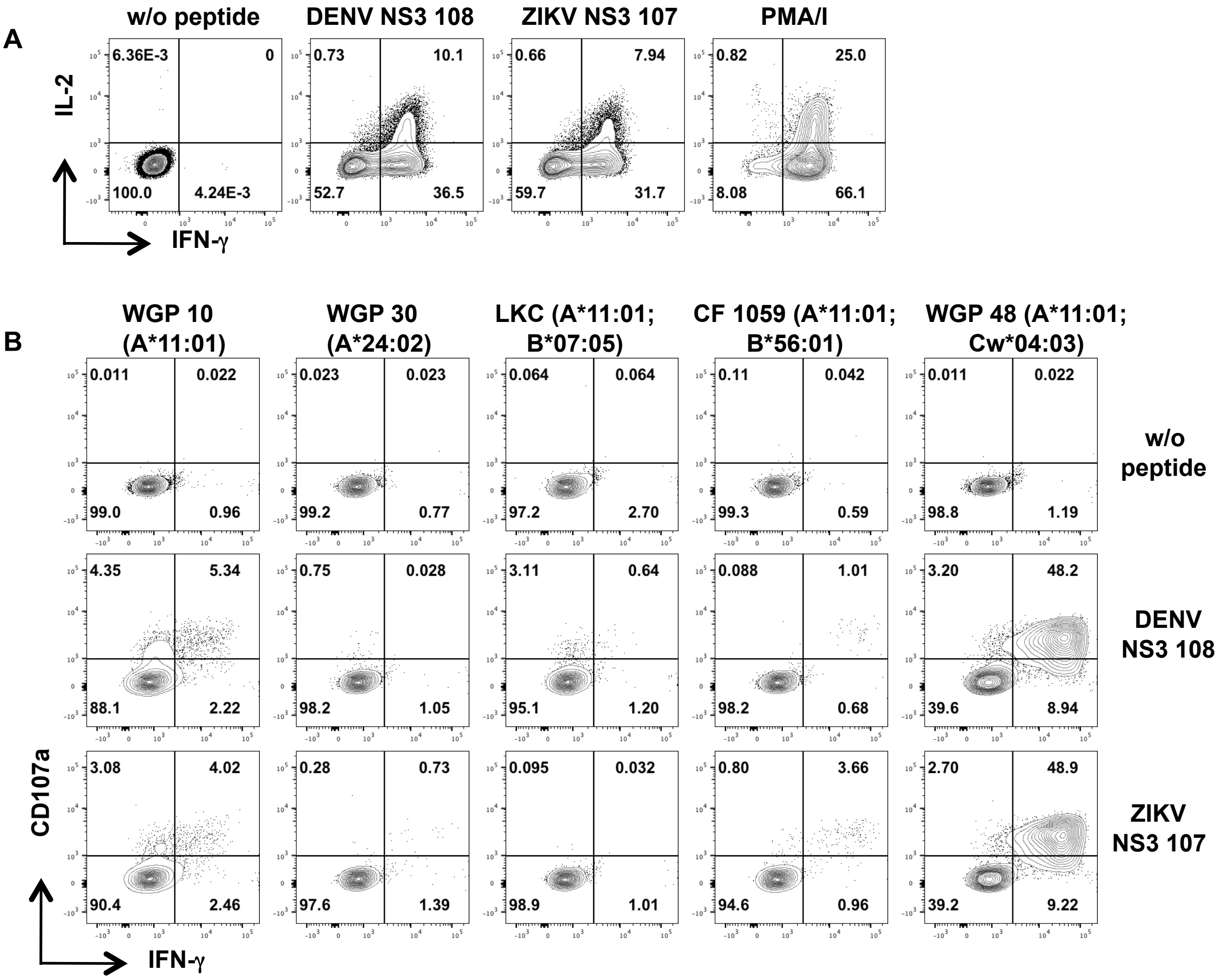

Supplementary Figure S2. Characterization of cross-reactive CD8+ T cells. (A) ICS showing IL-2 and IFN-γ production by CD8+ T cell lines stimulated with or without DENV NS3 108 and ZIKV NS3 107 peptides or with PMA/ionomycin. Plots are gated on live, CD8+ T cells. **(B)** Identification of the HLA class I restriction element of NS3 108/107 using EBV immortalized B cell lines (WGP 10, WGP 30, LKC, CF 1059 and WGP 48) matched with the subject for the indicated HLA type. Shown is production of CD107a and IFN-γ. Plots are gated on live, CD8+ T cells.

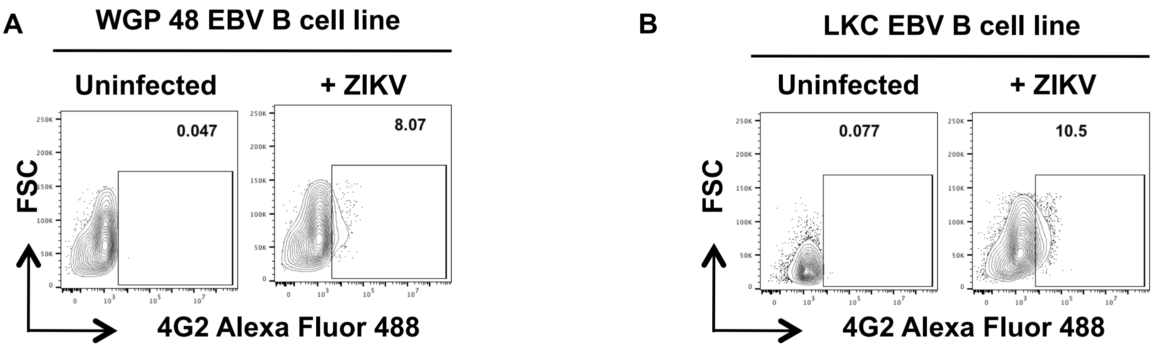

Supplementary Figure S3. Infection of EBV immortalized B cell lines with ZIKV. WGP 48 **(A)** or LKC **(B)** EBV immortalized B cell lines were infected with ZIKV strain MR 766 for 48 hours and subsequently stained with a mouse 4G2 antibody followed by an anti mouse IgG-Alexa Fluor 488 antibody for detection of ZIKV antigens.

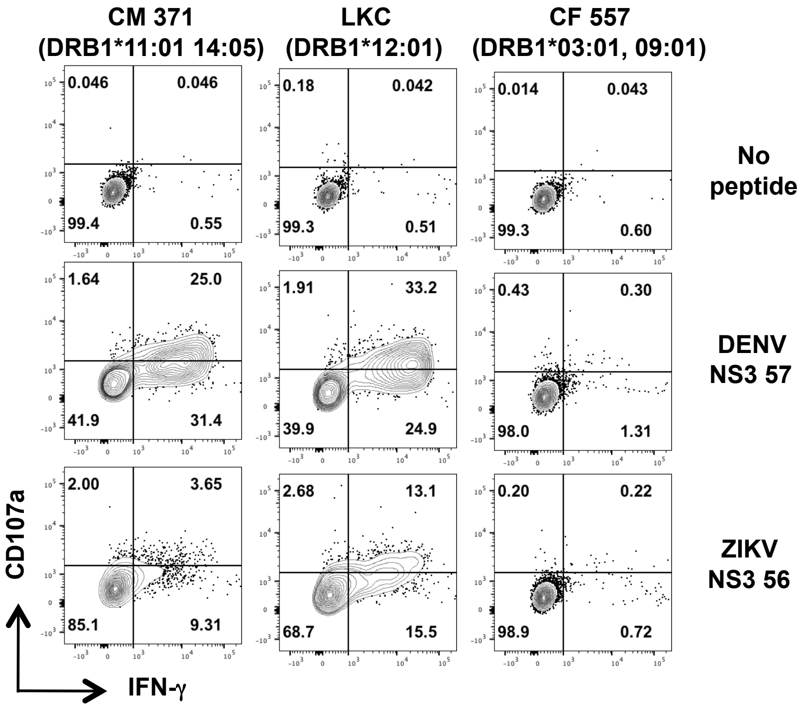

Supplementary Figure S4. Identification of an EBV immortalized B cell line that shares the MHC class II restriction element of DENV NS3 57/ ZIKV 56. The DRB1* alleles that are commonly expressed between the dengue-immune subject and the EBV cell lines are indicated. Shown is production of CD107a and IFN-γ. Plots are gated on live, CD4+ T cells.

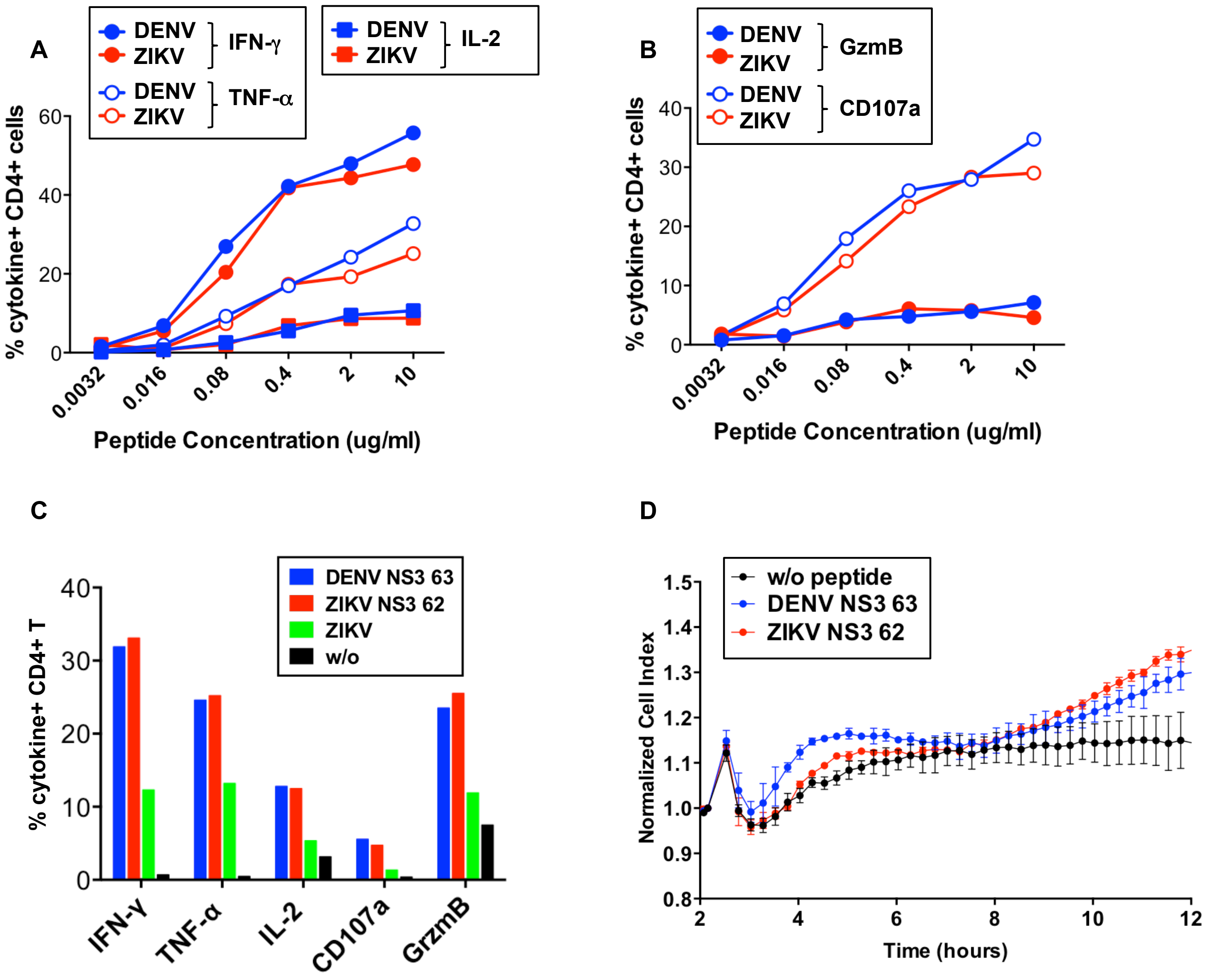

Supplementary Figure S5. Cytokine production and cytotoxic ability of the CD4+ T cell line specific for DENV NS3 63. (A, B) Production of effector cytokines **(A)** or cytolytic mediators **(B)** of the CD4+ T cell line after co-culture with autologous EBV-immortalized B cells pulsed with serial dilutions of DENV NS3 63 or ZIKV NS3 62 peptides, as assessed by ICS. **(C)** Production of IL-2, IFN-γ, TNF-α, granzyme B and CD107a by the DENV NS3 63 CD4+ T cell line upon overnight co-culture with autologous EBV-immortalized B cells that were pulsed with or without DENV NS3 63, ZIKV NS3 62 or were previously infected with ZIKV (strain MR766). **(D)** Kinetics of cellular lysis of autologous EBV cell lines pulsed with or without DENV NS3 63 or ZIKV NS3 62 measured by XCelligence RTCA.

Supplementary Tables SI A, B. Details of the dengue-immune (A) and dengue-naïve (B) subjects analysed in Figure 1.

A

Subject Code	Subject ID	Day from fever onset	Disease classification*	Primary/ Secondary DENV**	ZIKV IgG	ZIKV PRNT50
#1	Mab 60	70	DF NWS	ND	ND	ND
#2	Mab 86	60	DF NWS	Secondary	Positive	<20
#3	Mab 88	46	DF WS	Primary	Negative	<20
#4	Mab 113	59	DF WS	Primary	Negative	<20
#5	Mab 117	45	DF NWS	Primary	Negative	<20
#6	Mab 130	49	DF NWS	ND	ND	ND
#7	Mab 189	53	DF NWS	Secondary	Positive	<20
#8	Mab 190	46	DF WS	Primary	Negative	<20
#9	Mab 212	93	DF NWS	Secondary	Positive	<20
#10	Mab 216	51	DF WS	Primary	Negative	<20
#11	Mab 218	63	DF NWS	Primary	Negative	<20
#12	Mab 231	107	DF NWS	Primary	Negative	<20

B

Subject Code	DENV IgG	ZIKV IgG
HC 1	Negative	Negative
HC 2	Negative	Negative
HC 3	Negative	Negative
HC 4	Negative	Negative
HC 5	Negative	Negative
HC 6	Negative	Negative
HC 7	Negative	Negative
HC 8	Negative	Negative
HC 9	Negative	Negative
HC 10	Negative	Negative
HC 11	Negative	Negative
HC 12	Negative	Negative
HC 13	Negative	Negative
HC 14	Negative	Negative

*Dengue diagnosis was confirmed as described in Materials and Methods. DF WS and DF NWS indicates dengue fever with warning signs or dengue fever without warning signs, respectively.
** Secondary DENV infections are defined based on a DENV -specific IgM/IgG ratio of <1.8 in paired acute and convalescent plasma samples
ND = not determined; HC = healthy control
For CD4+ experiments: dengue-immune donors with a detectable CD4+ T cell response to DENV NS3/Capsid were included (all except #10, #12)
For CD8+ experiments: dengue-immune donors with a detectable CD8+ T cell response to DENV NS3/Capsid were included (all except #4, #9)

Supplementary Tables SII A, B. Details of the dengue-immune (A) and dengue-naïve (B) subjects analysed in Figure 2.

A

Subject code	Subject ID	Day from fever onset	Disease classification*	Primary/ Secondary DENV**	ZIKV IgG	ZIKV PRNT50	HLA-A	HLA-B	HLA-C	HLA-DRB1
#13	Mab 62	21	DF WS	Secondary	Positive	<20	A*11:01, A*24:02	B*07:05, B*56:01	Cw*04:03, Cw*07:07	DRB1*11:06, DRB1*15:02
#14	Mab 94	20	DF WS	Primary	Negative	<20	A*11:01, A*24:02	B*15:02, B*55:02	Cw*01:02, Cw*08:01	DRB1*08:01, DRB1*12:01
#15	Mab 101	28	DF WS	Secondary	Negative	<20	A*11:01, A*24:02	B*07:02, B*3901	Cw*07:02, Cw*07:02	DRB1*08:03, DRB1*15:01
#16	Mab 129	21	DF NWS	ND	ND	ND	A*11:01, A*24:02	B*13:02, B*40:06	Cw*06:02, Cw*08:01	DRB1*09:01, DRB1*09:01
#17	Mab 133	18	DF WS	ND	ND	ND	A*11:01, A*24:02	B*13:02, B*51:01	Cw*03:04, Cw*14:02	DRB1*12:02, DRB1*16:02
#18	Mab 150	14	DF NWS	Primary	Negative	<20	A*1101, A*11:01	B*15:01, B*58:01	Cw*03:02, Cw*03:03	DRB1*03:01, DRB1*14:05
#19	Mab 160	24	DF NWS	Primary	Negative	<20	A*11:01, A*24:02	B*15:01, B*58:01	Cw*03:02, Cw*03:03	DRB1*03:01, DRB1*19:01
#20	Mab 162	16	DF NWS	Secondary	Positive	<20	A*11:01, A*24:02	B*40:01, B*40:01	Cw*07:02, Cw*07:02	DRB1*09:01, DRB1*09:01
#9	Mab 212	93	DF NWS	Secondary	Positive	<20	ND	ND	ND	ND
#21	Mab 107	45	DF NWS	Secondary	Negative	<20	A*11:01 A*11:01	B*35:01 B*52:01	C*04:01 C*12:02	DRB1*04:03 DRB1*14:01

B

Subject code	DENV IgG	ZIKV IgG	HLA-A	HLA-B	HLA-C
HC 1	Negative	Negative	A*02:01, A*24:02	B*35:02, B*35:01	Cw*16:02, Cw*04:01
HC 2	Negative	Negative	A*11:01, A*02:01	B*35:01, B*35:01	Cw*04:01, Cw*04:01
HC 3	Negative	Negative	A*01:01, A*02:01	B*18:01, B*57:01	ND
HC 4	Negative	Negative	A*02:01, A*02:01	B*14:02, B*40:02	ND
HC 5	Negative	Negative	A*02:01, A*68:01	B*51:01, B*51:01	ND
HC 6	Negative	Negative	A*11:01, A*11:02	B*15:27, B*40:01	Cw*03:04, Cw*04:01
HC 7	Negative	Negative	A*11:01, A*24:02	B*18:01, B*44:02	Cw*05:01, Cw*07:01
HC 8	Negative	Negative	A*11:01, A*02:03	B*13:01, B*13:01	Cw*03:04, Cw*03:04
HC 9	Negative	Negative	A*11:01, A*24:02	B*13:01, B*40:01	ND
HC 10	Negative	Negative	ND	ND	ND
HC 11	Negative	Negative	ND	ND	ND
HC 12	Negative	Negative	ND	ND	ND
HC 13	Negative	Negative	A*11:01, A*33:03	B*35:01, B*58:01	ND
HC 14	Negative	Negative	ND	ND	ND

*Dengue diagnosis was confirmed as described in Materials and Methods. DF WS and DF NWS indicates dengue fever with warning signs or dengue fever without warning signs, respectively.
** Secondary DENV infections are defined based on a DENV -specific IgM/IgG ratio of <1.8 in paired acute and convalescent plasma samples
ND = not determined; HC = healthy control

Supplementary Table SIII. Details of the dengue-immune subjects analysed in Figure 3 (in addition to subjects from Supplementary Table SI A).

Subject Code	Subject ID	Day from fever onset	ZIKV PRNT50	Disease classification*
#22	Mab 214	62	<20	DF WS
#23	Mab 222	48	<20	DHF
#24	Mab 225	48	<20	DF WS

*Dengue diagnosis was confirmed as described in Materials and Methods. DF WS and DF NWS indicates dengue fever with warning signs or dengue fever without warning signs, respectively. DHF indicates dengue haemorrhagic fever

Supplementary Methods

Plaque Reduction Neutralization Test (PRNT). PRNT was performed on BHK-21 cells. Two-fold serial dilutions (1:20 to 1:320) of plasma from convalescent dengue patients were performed in media (RPMI, Gln, 2% FCS) and were incubated with 50 PFU of ZIKV (H/PF/2013 strain) in equal volumes for 1 hour before adding to BHK-21 cells. After 1 hour incubation at 37°C, media was aspirated and cells were overlaid with 1% methyl cellulose in media. After 5 days at 37°C, cells were fixed with 20% formaldehyde and stained with 1% crystal violet. Plaques were counted and the plasma concentration that provided 50% neutralization (PRNT50) was recorded (see Supplementary Tables SI a, II a and III).
